# Estrogen as Jekyll and Hyde: regulation of cell death

**DOI:** 10.12688/f1000research.4753.2

**Published:** 2014-09-29

**Authors:** Wen Zhou, Xiaoxia Zhu

**Affiliations:** 1 Braman Family Breast Cancer Institute, University of Miami Sylvester Comprehensive Cancer Center, University of Miami Miller School of Medicine, Miami, Fl 33136, USA; 2 Department of Biological Sciences, Columbia University, New York, 10027, USA; 3 Molecular Oncology Program, Division of Surgical Oncology, Dewitt Daughtry Family of Surgery, University of Miami Miller School of Medicine, Miami, Fl 33136, USA

## Abstract

Sustained estrogenic exposure increases the risk and/or the progression of various cancers, including those of the breast, endometrium and ovary. Unexpectedly, physiological level of estrogen together with a novel IKKα inhibitor BAY11-7082 could effectively induce cell apoptosis in ER-positive breast cancer cells, suggesting combining estrogen with IKKα inhibition may be beneficial for breast cancer patients. This opinion article touches upon the dual role estrogen played in inducing cancer cell death and asks whether use of estrogen in combination with IKKα-targeted therapy would be possible reconsider the newly identified crosstalk between ER and NFκB pathway which can be utilized to switch the effects of estrogen on cell death.

## Introduction

Our research projects currently focus on the understanding of the interplay between different signaling cascades and estrogen receptor alpha (ER) dependent transcription activation in breast cancer. ER is a master transcription factor frequently elevated in breast cancer patients. ER-positive breast cancers usually occur in up to two thirds of newly diagnosed breast cancer patients. Drugs (such as the antiestrogens tamoxifen and raloxifene) have been very effective in treating ER-positive breast cancers and have saved millions of lives. However, this standard treatment does not work for all ER-positive breast cancer patients. Therefore, we enthusiastically review recent research breakthroughs in the context of our ever-developing understanding on ER function. These new mechanistic insights on the crosstalk between ER and NFκB pathway will lay the ground work for improved clinical approaches to the treatment of this type of breast cancer.

For many years, those of us working in the field were used to looking at estrogen as a mitogen through its genomic function mediated by ER-dependent transcription program. Now we realize that estrogen through ER, in addition to regulating gene expression, crosstalks with many non-genomic signaling pathways involved in cell growth, differentiation and apoptosis. These newly identified interplays may give a different flavor to long conceived mitogenic role of estrogen. In this opinion article, we generally reviewed the changing attitude toward the use of estrogen in the clinic, with a focus on newly discovered pro-apoptotic roles of estrogen when combined with IKKα inhibitors. We also discussed the possibility of estrogen and IKKα inhibitor dual-therapy in ER-positive breast cancer treatment.

## Historical perspectives: changing attitudes toward the use of estrogen in the clinic

Sir George Thomas Beatson (1896) use oophorectomy to reduce estrogen level in premenopausal women in order to prevent breast cancer occurrence. It was for the first time to reveal the mitogenic role of estrogen at physiological concentration ^1^. Half a century later, Haddow *et al*. (1944) firstly used a high-dose estrogen to induce tumor regression of hormone-dependent breast cancer in postmenopausal women ^2^. Therefore, the effect of estrogen is dose-dependent, and thus it is important to take into account the hormonal status of women (pre- or postmenopausal). Huggins *et al.* (1952) pioneered adrenalectomy to reduce estrogen level for treating mammary cancer ^3^. Huggins’ work is internationally recognized by the most prestigious Nobel Prize (1966) for the contribution in the development of endocrine therapy in hormone-regulated cancer. Elwood Vernon Jensen (1958) characterized the first receptor for estrogen - estrogen receptor alpha. Soon after these discoveries, extensive mechanistic studies have gained large information about estrogen’s physiological functions and carcinogenic roles. The Women’s Health Initiative research program (1991) initiated a 15-year study enrolled 161,808 generally healthy women aged 50–79 to evaluate the beneficial effects of postmenopausal hormone replacement therapy on heart diseases, bone fractures, and cancers ^4^. Due to the increased incidence of breast cancer, stroke, and cardiovascular complications in women treated with estrogen alone or with a combination of estrogen and progesterone, the study was terminated prematurely in 2002. Though extensively studied, the definite understanding of the mechanism of estrogen action always challenges our mind.

## The challenge: paradoxical role of estrogen in cell death

Estrogen regulates the proliferation and development of tissues expressing estrogen receptors and ER is mainly expressed in breast epithelium, ovary and endometrium. Thus, estrogen is mitogenic for cultured ER positive breast cancer lines. The mitogenic effects of estrogen at the G1-to-S transition are mediated by the key effectors of estrogen action, c-Myc, cyclin D1 and E2F-1 ^5– 7^. c-Myc expression occurs within 15 min of estrogen stimulation, among the earliest responses to estrogen. Estrogen also rapidly induces cyclin D1 expression. In the G1 phase, estrogen drives E2F-1 expression. Nongenomically, Estrogen binding to the ER stimulates rapid activation of Src and signaling pathways MAPK and PI3K/Akt pathways that affect cell survival ^8, 9^. Based on these understandings of estrogen action, ER protein is assayed in newly diagnosed breast cancers because it is a clinically useful prognostic factor and predicts responsiveness to ER blocking drugs such as Tamoxifen.

Paradoxically, estrogen induces apoptosis under certain circumstances. As aforementioned, high-dose estrogen was used to induce tumor regression of hormone-dependent breast cancer in postmenopausal women before the introduction of tamoxifen ^2^. This regimen is of clinical interest, given that long-term treatment of breast cancer with the antiestrogen tamoxifen often leads to drug resistance and that sustained tamoxifen exposure may sensitize breast cancer cells to estrogen therapy ^10^. The field of the mysterious dual effects of estrogen on apoptosis has not much progress until recently.

## Research breakthrough

Perillo Group from the Second University of Naples identified a key player, IKKα in the switch of estrogen action in apoptosis ^11^. They found ER agonist 17β-estradiol (E2) and IKKα kinase specific inhibitor BAY11-7082 (BAY) in combination can induce apoptosis in ERα positive breast cancer cell line, and dual-therapy now receives more and more attention.

**Figure 1.**
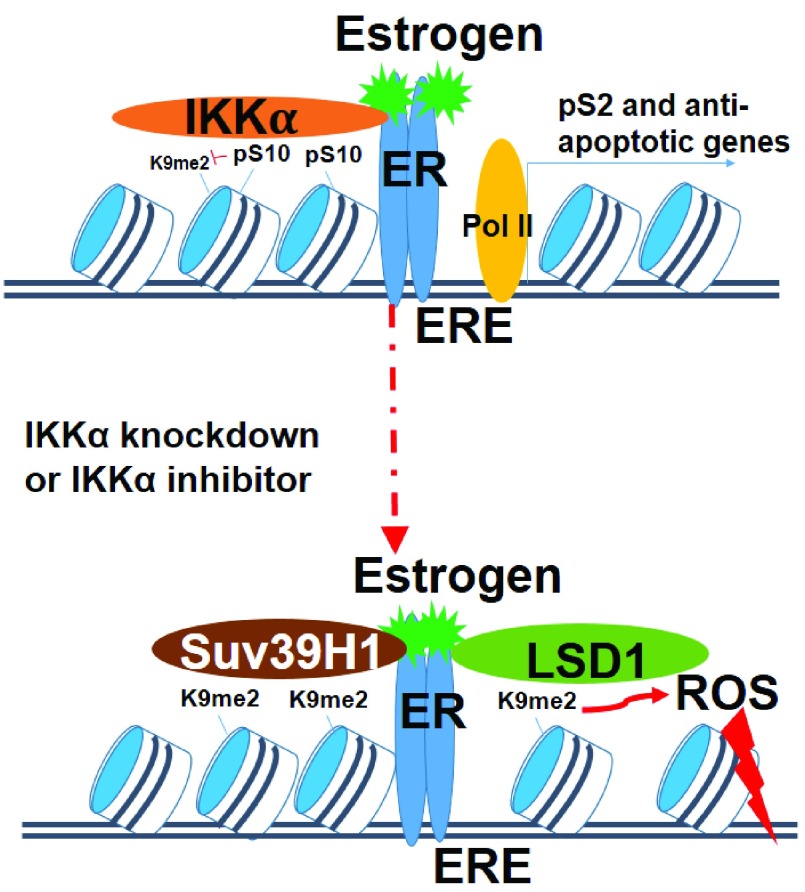
Involvement of IKKα in estrogen-triggered ER-dependent activation of the *pS2* promoter. Upper panel: chromatin landscape and factors present at the *pS2* (as known as *TFF1*) promoter in the presence of IKKα. The pS2 promoter is enriched with nucleosomes (blue and white cylinders) that dwell in positions proximal to the transcription start site (+1 position) and at ER binding sites. Only low levels of histone H3 lysine 9 dimethylation (H3K9me2) exist due to the space repulsion of histone methyltransferase from IKKα residing at the neighboring H3S10 site. RNA polymerase holoenzyme (Pol II) (yellow oval) is present at the proximal promoter region near the transcription start site (TSS, shown by black vertical line) of the pS2 gene. Lower panel: chromatin landscape and factors present at pS2 promoter following IKKα knockdown or IKKα inhibitor BAY11-7082 treatment. Once levels of IKKα have decreased, ER recruits the histone methyltransferase Suv39H1 or demethylase LSD1 proteins to bind within the *pS2* promoter. Once the LSD1 is activated and demethylates its target H3K9me2, it generates reactive oxygen species (ROS) to cause DNA damage effects including base oxidation and nicks results from DNA damage itself and related DNA repair. In sum, the inhibition of IKKα results in the reversion of estrogen triggered anti-apoptotic effects to pro-apoptotic effects.

In the journal Cell Death & Differentiation, Perillo *et al.* recently reported that the inhibition of IKKα by BAY switched the effect of estrogens on breast cancer cells from anti- to pro-apoptotic, which leads the exploration of therapeutic benefits of estrogen into a new era ^11^. IKKα is the kinase responsible for histone H3 Ser 10 phosphorylation (H3pS10) ^12^. H3pS10 is known to inhibit H3 Lys 9 dimethylation (H3K9me2) in a space repulsion model ^13^. Thus, inhibiting H3pS10 by targeting IKKα facilitates estrogen-triggered ER-dependent recruitment of histone methyltransferase Suv39H1. Histone demethylase LSD1 demethylating the Suv39H1 target sites H3K9me2 was increased concomitantly. LSD1-mediated demethylation process is known to produce reactive oxygen species (ROS) and cause ROS-mediated DNA damaging effects ^14^. The net results after IKKα knowndown is causing more DNA damages to cancer cells through estrogen triggered ER-dependent Suv39H1 and LSD1 binding to ER target gene promoter ( Figure 1).

In short, Perillo group identifies a novel crosstalk between IKKα and estrogen signaling and shows that inhibition of IKKα-mediated histone phosphorylation switch ER-mediated anti-apoptotic effects to ER-dependent ROS-mediated breast cell death, which implicates potential dual-therapy of ER agonist (E2) together with IKKα inhibitor (BAY) in a variety of hormone-regulated cancers. Questions remain as to whether this study using tissue-culture adapted cell lines such as MCF-7 is relevant to clinic practice. The consideration here is mainly based on the well documented limitations of using established cell lines to predict a clinical response, with several compounds that show dramatic tumor killing in cell models failing to yield clinical benefits. Given that established cell lines do not fully reflect the genomic heterogeneity we now appreciate existing in different breast tumors, it will be important to test the same drug combination in a more clinical-relevant sophisticated tumor-stromal microenvironment. This merits further investigation using co-culture between malignant epithelial cells, fat and vascular and inflammatory cells as this likely to influence response to therapy. Additionally, *in vivo* studies employing patient-derived xenograft model would be very informative in promoting these novel ideas toward clinical trials. Recognizing the known complication of translating scientific discovery to clinical benefit, the question of whether inhibition of IKKα is a valuable therapeutic strategy in ER-positive breast cancer will have to await more positive results from other model systems and also the further development of additional IKKα inhibitors with minimal potential toxicity and highly bioavailable.

## Future perspectives

In the last few years, there have been significant shifts in the attitudes toward the use of estrogen in clinic. Estrogen exhibits a broad range of functions that regulates cell proliferation and homeostasis in many tissues. Despite beneficial estrogen functions, sustained estrogenic exposure increases the risk and/or the progression of various cancers, including those of the breast, endometrium and ovary ^15^. The International Agency for Research on Cancer (IARC) has listed estrogen as known human carcinogen ^16^. Now, the success of the combination of E2 and BAY will certainly become an “accelerator” to the alternative use of estrogen in treating cancers and we expect to see more positive pre-clinical and/or clinical results on BAY or other IKKα inhibitors in ER-positive hormone responsive cancers in the near future.
