# Supplementary material for: Estrogen as Jekyll and Hyde: regulation of cell death
Source: F1000Res. 2014 Sep 29;3:161. Originally published 2014 Jul 11. [Version 2] doi: 10.12688/f1000research.4753.2 (PMC4243742; doi:10.12688/f1000research.4753.2)
Supplement: Peer review history

# Peer review: Referee response for version 2

**DOI:** 10.5256/f1000research.5741.r6796

**Published:** 2014-11-24

**Authors:** Steven L Young

**Affiliations:** 1 Department of Obstetrics & Gynecology, University of North Carolina at Chapel Hill, Chapel Hill, NC, USA

The authors have addressed all my concerns.

I have read this submission. I believe that I have an appropriate level of expertise to confirm that it is of an acceptable scientific standard.

# Peer review: Referee response for version 2

**DOI:** 10.5256/f1000research.5741.r6389

**Published:** 2014-10-13

**Authors:** Philippa Saunders

**Affiliations:** 1 Center for Reproductive Health, University of Edinburgh, Edinburgh, UK

The authors have provided responses to my original comments and incorporated some changes into their manuscript which have improved it.

**Suggestions for minor modifications**:

Section entitled ‘Challenges’

The authors state that estrogen regulates the proliferation and development of tissues expressing estrogen receptors and that ‘ER’ is expressed in steroid--‐ responsive female tissues such as the ovary, endometrium and breast. The authors further state ‘Thus, estrogen is mitogenic for cultured ER positive breast cancer cell lines.”

The authors need to amend this paragraph to make it clear that there are two estrogen receptor genes both of which are expressed in breast cancers ^1^^-^^3^. Studies on knockout mice have highlighted that expression of the ERα isoform has been identified as critically important to cell proliferation in the uterus and breast ^4^^,^^5^. Studies in breast cancer cell lines have highlighted the importance of ERα in both up and down-regulation of cell proliferation and this remains a very active area of research ^6^^,^^7^. The authors are encouraged to amend the text of this section changing ‘ER’ to ERα to make better sense of comments made in the section entitled ‘Research Breakthrough’.

I have read this submission. I believe that I have an appropriate level of expertise to confirm that it is of an acceptable scientific standard.

## References

[1] GustafssonJAWarnerM: Estrogen receptor beta in the breast: role in estrogen responsiveness and development of breast cancer. *J Steriod Biochem Mol Biol*.2000;74(5) : 161245-24810.1016/S0960-0760(00)00130-8Reference source11162931

[2] SpeirsVParkesATKerinMJWaltonDSCarletonPJFoxJNAtkinSL: Coexpression of estrogen receptor alpha and beta: poor prognostic factors in human breast cancer. *Cancer Res*.1999;59(3) :525-528Reference source9973193

[3] ShaabanAMGreenARKarthikSAlizadehYHughesTAHarkinsLEllisIORobertsonJFPaishECSaundersPTGroomeNPSpeirsV: Nuclear and cytoplasmic expression of ERbeta1, ERbeta2, and ERbeta5 identifies distinct prognostic outcome for breast cancer patients.*Clin Cancer Res*.2008;14(16) : 1615228-5523510.1158/1078-0432.CCR-07-4528Reference source18698041

[4] HewittSCKorachKS: Oestrogen receptor knockout mice: roles for oestrogen receptors alpha and beta in reproductive tissues.*Reproduction*.2003;125(2) : 161143-14910.1530/rep.0.1250143Reference source12578528

[5] CouseJFKorachKS: Estrogen receptor null mice: what have we learned and where will they lead us?. *Endrocr Rev*.1999;20(3) :358-417Reference source1036877610.1210/edrv.20.3.0370

[6] StenderJDFrasorJKommBChangKCKrausWLKatzenellenbogenBS: Estrogen-­‐regulated gene networks in human breast cancer cells: involvement of E2F1 in the regulation of cell proliferation. *Mol Endocrinol*.2007;21(9) : 1612112-212310.1210/me.2006-0474Reference source17550982

[7] RosellMNevedomskayaEStellooSNautiyalJPoliandriASteelJHWesselsLFCarrollJSParkerMGZwartW: Complex Formation and Function of Estrogen Receptor alpha in Transcription Requires RIP140. *Cancer Res*.2014;74(19) : 1615469-547910.1158/0008-5472.CAN-13-3429Reference source25145671

# Peer review: Referee response for version 1

**DOI:** 10.5256/f1000research.5075.r6196

**Published:** 2014-09-22

**Authors:** Philippa Saunders

**Affiliations:** 1 Center for Reproductive Health, University of Edinburgh, Edinburgh, UK

New ideas about the way oestrogens might affect the growth, differentiation and functional development of cancers, including those of the breast, are always welcome. The authors' Opinion Article questions whether giving oestrogens (rather than the more common anti-oestrogens) may merit exploration as an alternative treatment for breast cancers when provided in combination with agents that alter inflammatory signalling pathways. A limitation of the commentary is that it has focused on data from studies using breast cancer cell lines such as the MCF-7 cells cited in the studies by the Perillo group ^1^^-^^3^. A limitation of all studies using tissue-culture adapted cell lines such as MCF-7 is that they do not reflect the genomic heterogeneity we now appreciate exists in different breast tumours ^4^, only a proportion of which are considered classically activated by oestrogens because they are ER alpha positive. In spite of these reservations the authors do raise some interesting points.

Firstly, they highlight the potential for inflammatory mediators such as those that activate the NFκB signalling pathway as targets for therapies in breast cancer treatment. One example provided is the data from the Perillo group, reporting that inhibition of IKKα kinase specific inhibitor BAY11-7082 might be beneficial in switching the effects of oestrogens from anti- to pro-apoptotic. These studies are in agreement with those from a number of other authors claiming that combination therapies are likely to be beneficial in treatment of this malignancy. Notably, there is an interesting paper by Biswas *et al.* published in 2004 ^5^ which examines how NFκB activation in breast cancer specimens might have a role in cell proliferation and apoptosis. Notably, in the Biswas paper, they report results suggesting that activated NFκB is predominantly detected in ER *negative *rather than ER positive breast tumours. This paper, as well as the recent genomic proflling of breast cancers from 2000 patients ^4^ highlights the importance of considering the both the immediate microenvironment of the tumour as well as the adjacent non-tumour tissue. Notably, in considering how oestrogen therapy might be utilised, it is important to take into account the hormonal status of the woman (pre or post menopausal) as well as the compelling data that has demonstrated intra-tissue oestrogen biosynthesis in fat and other cells close to the malignant epithelial cells *in situ* ^6^. A paper that has just appeared in the Journal of Clinical Endocrinology and Metabolism by Savolainen-Peltonen and colleagues ^7^ is particularly relevant. In this paper, the authors compared the metabolic pathways producing active oestradiol in breast subcutaneous adipose tissue of postmenopausal women with and without cancer, and these showed there were differences depending on whether the adipose tissue was proximal or distal to a tumour. If the tumour is already bathed in high concentrations of oestrogen either from the periphery or local biosynthesis then addition of more ligand is unlikely to be effective.

The local inflammatory environment of the tumour is an important factor in tumour progression and it is influenced by activation of NFκB activity leading to production of inflammatory mediators such as TNFα, IL-1β and prostaglandin E2 from adipocyteassociated macrophages and COX-2 inhibitors have been cited as effective in reducing recurrence of cancer ^8^. Local increases in inflammatory mediators in turn stimulate adipocyte aromatase expression/activity and hence oestradiol production ^9^ that may be particularly relevant to obese women ^9^^,^^10^. Thus, when considering suppression of NFκB activity in combination therapy there may be many context-dependent impacts on the tumour microenvironment to consider.

The role of NFκB activation as a regulator of inflammatory signalling is particularly important to consider in the context of endocrine resistant breast cancer which cannot be addressed using MCF-7 cells. A new paper in Molecular Cellular Endocrinology by Litchfield *et al.* ^11^ notes that regulation of endocrine-resistant breast cancer cells may also be mediated by other transcription factors such as COUPTFII. So the keys questions to be addressed in considering the value of the future prospective advanced within this Opinion piece are the following:

1. Are studies in isolated cell lines such as the one described really relevant when it comes to translating new therapies into practice?
2. Is inhibition of inflammatory mediators such as that using the BAY compound really most effective in cells that are not actually estrogen responsive?This merits further investigation.
3. Finally, how can we move forward with the field?

 

In our opinion, an excellent way to advance these studies would be to conduct further investigations using methods which better reflect the microenvironment of an intact tumour *in situ*, this should include studies on the interplay between malignant epithelial cells, fat and vascular and inflammatory cells as this likely to influence response to therapy and it cannot be under-estimated that the individual responses to oestrogens by these different cell types may vary considerably. Examples of such studies currently include 3 being explored in the context of breast cancer, including organoids, tissue slices or xenografts. These approaches are more likely than studies in MCF-7 cells to inform choice of new combination therapies for testing in clinical trials; oestrogen combined with NFκB inhibition should be tested in these systems ^12^^-^^14^.

I have read this submission. I believe that I have an appropriate level of expertise to confirm that it is of an acceptable scientific standard, however I have significant reservations, as outlined above.

## References

[1] PerilloBDi SantiACerneraGOmbraMNCastoriaGMigliaccioA: Phosphorylation of H3 serine 10 by IKKα governs cyclical production of ROS in estrogen-induced transcription and ensures DNA wholeness.*Cell Death Differ*.2014;21(9) : 1611503-150310.1038/cdd.2014.91Reference source24971480PMC4131185

[2] PerilloBDi SantiACerneraGOmbraMNCastoriaGMigliaccioA: Nuclear receptor-induced transcription is driven by spatially and timely restricted waves of ROS: The role of Akt, IKKα, and DNA damage repair enzymes.*Nucleus*.2014;5(5) : 16110.4161/nucl.36274Reference source25482200PMC4164490

[3] PerilloBOmbraMNBertoniACuozzoCSacchettiSSassoAChiariottiLMalorniAAbbondanzaCAvvedimentoEV: DNA oxidation as triggered by H3K9me2 demethylation drives estrogen-induced gene expression.*Science*.2008;319(5860) : 161202-20610.1126/science.1147674Reference source18187655

[4] CurtisCShahSPChinSFTurashviliGRuedaOMDunningMJSpeedDLynchAGSamarajiwaSYuanYGrafSHaGHaffariGBashashatiARussellRMcKinneySMETABRIC Group: The genomic and transcriptomic architecture of 2,000 breast tumours reveals novel subgroups. *Nature*.2012;486(7403) : 161346-35210.1038/nature10983Reference source22522925PMC3440846

[5] BiswasDKShiQBailySStricklandIGhoshSPardeeABIglehartJD: NF-kappa B activation in human breast cancer specimens and its role in cell proliferation and apoptosis. *Proc Natl Acad Sci U S A*.2004;101(27) : 16110137-1014210.1073/pnas.0403621101Reference source15220474PMC454178

[6] SimpsonHWCandlishWPausonAWMcArdleCSGriffithsKSmallRG: Genesis of breast cancer is in the premenopause. *Lancet*.1988;2(8602) :74-76Reference source289870110.1016/s0140-6736(88)90007-4

[7] Savolainen-PeltonenHVihmaVLeideniusMWangFTurpeinenUHamalainenETikkanenMJMikkolaTS: Breast Adipose Tissue Estrogen Metabolism in Postmenopausal Women with or without Breast Cancer. *J Clin Endocrinol Metab*.2014; Reference source2521555910.1210/jc.2014-2550

[8] BowersLWMaximoIXBrennerAJBeeramMHurstingSDPriceRSTekmalRRJollyCAdeGraffenriedLA: NSAID Use Reduces Breast Cancer Recurrence in Overweight and Obese Women: Role of Prostaglandin-Aromatase Interactions.*Cancer Res*.2014;74(16) : 1614446-445710.1158/0008-5472.CAN-13-3603Reference source25125682PMC12935481

[9] SubbaramaiahKHudisCChangSHHlaTDannenbergAJ: EP2 and EP4 receptors regulate aromatase expression in human adipocytes and breast cancer cells. Evidence of a BRCA1 and p300 exchange.*J Biol Chem*.2007;283(6) : 1613433-344410.1074/jbc.M705409200Reference source18083712

[10] SubbaramaiahKHoweLRPortERBrogiEFishmanJLiuCHHlaTHudisCDannenbergAJ: HER-2/neu status is a determinant of mammary aromatase activity in vivo: evidence for a cyclooxygenase-2-dependent mechanism.*Cancer Res*.2006;66(10) : 1615504-551110.1158/0008-5472.CAN-05-4076Reference source16707480

[11] LitchfieldLMAppanaSNDattaSKlingeCM: COUP-TFII inhibits NFkappaB activation in endocrine-resistant breast cancer cells. *Mol Cell Endocrinol*.2014;382(1) : 161358-36710.1016/j.mce.2013.10.010Reference source24141032PMC5089806

[12] WalshAJCookRSSandersMEAurisicchioLCilibertoGArteagaCLSkalaMC: Quantitative optical imaging of primary tumor organoid metabolism predicts drug response in breast cancer.*Cancer Res*.2014;74(18) : 1615184-519410.1158/0008-5472.CAN-14-0663Reference source25100563PMC4167558

[13] SunXMaoYWangJZuLHaoMChengGQuQCuiDKellerETChenXShenKWangJ: IL-6 secreted by cancer-associated fibroblasts induces tamoxifen resistance in luminal breast cancer. *Oncogene*.2014;33(35) : 161445010.1038/onc.2014.224Reference source24909173

[14] HollidayDLMossMAPollockSLaneSShaabanAMMillican-SlaterRNashCHanbyAMSpeirsV: The practicalities of using tissue slices as preclinical organotypic breast cancer models. *J Clin Pathol*.2013;66(3) :253-255Reference source2307639610.1136/jclinpath-2012-201147

# Peer review: Referee response for version 1

**DOI:** 10.5256/f1000research.5075.r5459

**Published:** 2014-08-11

**Authors:** Steven L Young

**Affiliations:** 1 Department of Obstetrics & Gynecology, University of North Carolina at Chapel Hill, Chapel Hill, NC, USA

In this opinion manuscript, Zhou and Zhu highlight recent research that suggests estrogen can act on opposing signaling pathways to either promote proliferation or cell death in breast cancer cells and, further, that the signaling can be biased toward cell death by inhibition of IKKa action. The manuscript is thought provoking and timely and the argument is logical and generally well-constructed. The title is eye-catching and appropriate. The figure is attractive and highlights the key point of the manuscript. If the areas of concern (below) are addressed, the conclusions will be justified by the argument presented.

I have four areas of concern:

1. The manuscript is marred by frequent grammatical and English language usage errors as well as occasional typographic errors. While readable, it is sometimes difficult to determine the precise meaning of the authors’ statements. 
2. In the introduction, the authors state that estrogen, in combination with IKKa, can induce breast cancer apoptosis. However, it is IKKa inhibition that is used, suggesting that it is the absence of IKKa effect that is important - this should be clarified as currently the penultimate sentence of the introduction paragraph appears to be contradicted by the sentence following.
3. The authors propose that IKKa inhibition has great promise for clinical use, but fail to remark on factors that would be important for feasibility of such therapy (see below):
   
  1. If IKKa were inhibited systemically, would apoptosis be largely limited to the cancer cells? What about other estrogen target tissues?
  2. Are there known toxicities of BAY or other IKKa inhibitors that would need to be considered? 
  3.  Are the concentrations of estradiol and IKKa inhibitor necessary to induce cell death achievable in humans?
  4. Are the concentrations of estradiol required for induction of cell death in the normal pre-menopausal range, or are these pharmacological levels: important implications for treatment of women with functioning ovaries and for the thrombogenic side effects of estrogen therapy, which are dose-related.

4. The abstract does not effectively summarize the manuscript.

I have read this submission. I believe that I have an appropriate level of expertise to confirm that it is of an acceptable scientific standard, however I have significant reservations, as outlined above.
